# Heavy metal removal using an advanced removal method to obtain recyclable paper incineration ash

**DOI:** 10.1038/s41598-022-16486-8

**Published:** 2022-07-27

**Authors:** Hak-Min Kim, Tae-Yeol Choi, Min-Ju Park, Dae-Woon Jeong

**Affiliations:** 1 Industrial Technology Research Center, Changwon National University, 20 Changwondaehak-ro, Changwon, Gyeongnam 51140 Republic of Korea; 2 Department of Smart Environmental Energy Engineering, Changwon National University, 20 Changwondaehak-ro, Changwon, Gyeongnam 51140 Republic of Korea; 3 Department of Environmental & Energy Engineering, Changwon National University, 20 Changwondaehak-ro, Changwon, Gyeongnam 51140 Republic of Korea

**Keywords:** Chemistry, Engineering

## Abstract

Various agents, including ethylenediaminetetraacetic acid, oxalic acid, citric acid, and HCl, were applied to remove heavy metals from raw paper incineration ash and render the ash recyclable. Among these prepared agent solutions, ethylenediaminetetraacetic acid showed the highest efficiency for Pb removal, while oxalic acid showed the highest efficiencies for Cu, Cd, and As removal. Additionally, three modes of an advanced removal method, which involved the use of both ethylenediaminetetraacetic acid and oxalic acid, were considered for use at the end of the rendering process. Among these three modes of the advanced removal method, that which involved the simultaneous use of ethylenediaminetetraacetic acid and oxalic acid, i.e., a mixture of both solutions, showed the best heavy metal removal efficiencies. In detail, 11.9% of Cd, 10% of Hg, 28.42% of As, 31.29% of Cu, and 49.19% of Pb were removed when this method was used. Furthermore, the application of these three modes of the advanced removal method resulted in a decrease in the amounts of heavy metals eluted and brought about an increase in the CaO content of the treated incineration ash, while decreasing its Cl content. These combined results enhanced the solidification effect of the treated incineration ash. Thus, it was confirmed that the advanced removal method is a promising strategy by which recyclable paper incineration ash can be obtained.

## Introduction

One of the major concerns of the manufacturing industry with regard to sustainability, which includes economic, social, and environmental aspects, is waste removal^1^. In particular, there has been a significant increase in the amount of paper waste generated owing to the increasing popularity of the use of paper, mainly cardboard, as packaging material in e-commerce^2^. In fact, in 2016, the European Union alone produced 87.5 million metric tons of packaging waste, of which 35.4 million metric tons consisted of paper and cardboard. Similarly, in 2017, the United States generated approximately 61 million metric tons of packaging waste^2^. Therefore, it is expected that the production of paper and cardboard will reach 700–900 million metric tons by 2050, which will result in the generation of approximately 400 million tons of paper waste^3^. While several strategies for the reuse of paper sludge are actively being investigated, large amounts are still being disposed of in landfills, with significant negative economic and environmental impacts^4–6^.

Paper waste incineration is an effective method that can be employed to recycle paper waste and minimize the burden of its disposal^7–9^. The ash generated during the incineration procedure could be recycled and used for various purposes, such as lightweight aggregates for road construction, asphalt or concrete filling, construction materials, and as a solidification agent; these applications are possible because the ash contains lime, silica, and alumina^10–15^. In particular, the high calcium oxide content (40–70%) of the ash generated from paper waste incineration plays an important role in improving the compressive strength of supplementary cementitious materials^11,16–20^. However, for paper incineration ash to be used in this regard, the removal of heavy metals, which are associated with serious health hazards, even in small quantities, is necessary^21,22^. The heavy metals causes the critical diseases for the human^23,24^. In the case of Pb, it causes the death or damage to the nervous system, brain, and kidneys^24,25^. Chrome can damage to kidneys, liver, circulatory, and nervous system^26–28^. The inorganic forms of mercury causes spontaneous abortion, congenital malformation and gastrointestinal disorders^24,29^. In addition, this mercury attributes to erethism, acrodynia, gingivitis, stomatitis, neurological disorders, and total damage to the brain^23^. Arsenic is one of the most dangerous heavy metal, causing death and diseases in nerve system^23,30^. That is, heavy metal stabilization or removal is required prior to the application of incineration ash as a supplementary cementitious material^31,32^. Furthermore, the removal of heavy metals by washing offers great advantages in the recycling of paper incineration ash given the ease of the washing operation and the fact that it results in the permanent removal of heavy metals^33,34^. However, the investigation of washing operation is required because the studies for the removal of heavy metals in the paper incineration ash are not enough. In this regard, several solutions have been applied in the washing of incineration ash with the successful removal of heavy metal species^35–40^. In particular, the chelate functional groups in EDTA can remove heavy metals by complexing the heavy metal species^41,42^. Organic acids such as oxalic acid and citric acid can promote the desorption of heavy metals from ash^43,44^. In addition, when using organic acids, the reductive conditions generated in solution inhibit the conversion of heavy metal ions to heavy metals on the surface of the ash. HCl provides a large amount of hydrogen ions, which converts the heavy metals on the surface of the ash to heavy metal ions. It has been observed that HCl can be used to effectively remove heavy metals, such as Pb, Cu, and Cd, from waste activated sludge^35^. However, the chlorine present in the HCl solution can cause environmental problems^36^. In contrast, organic acids, with a lesser environmental impact, have also shown excellent performance with respect to the removal of heavy metals from soils or ash. Specifically, Hong et al. reported that more than 70% of Pb and Cd can be removed using 0.1 M ethylenediaminetetraacetic acid (EDTA) solution and citric acid (CA), while Shi et al. reported soil Cd and Pb removal rates of 73.1 and 98.0%, respectively^37,38^. It has also been reported that oxalic acid (OA) is effective in the removal of As, showing a removal efficiency of more than 50% for As^39^.

In most studies in which different agents have been used to realize heavy metal removal, the focus has always been on the application of single-agent solutions. However, the various agents studied to date show different removal efficiencies for different heavy metals^35–38,45^. This implies that effectively realizing the removal of multiple heavy metals using a single-agent solution remains a challenge. Therefore, in this study, we considered an advanced removal method involving a combination of two agents, yielding an increased performance when employed to realize heavy metal removal compared to when the same agents are used individually. It is expected that this study provides the new approach for the improved removal efficiencies of heavy metals in paper incineration ash. Synergy effects are expected when two kinds of agents that have different removal effects are used. To identify two agents with higher heavy metal removal efficiencies for use in combination, EDTA, CA, OA, and HCl were used as single-agent solutions, and their heavy removal efficiencies were analyzed by investigating the composition of treated paper incineration ash after their use as removal solutions. The removal efficiencies was calculated based on the heavy metal contents in paper incineration ash which confirmed ICP-OES, Mercury analyzer, and UV–visible spectroscopy system. Thereafter, to optimize the sequence of removal using the selected agents, three modes of the advanced removal method were investigated. Furthermore, the possibility for recycling and utilizing the treated incineration ash was examined by analyzing its calcium oxide and chlorine contents after removal, given that calcium oxide and chlorine promote and downgrade the compressive strength of supplementary cementitious materials, respectively^18,46^. The contents of calcium oxide and chlorine in the paper incineration ash were measured using XRF analysis. As a result, the goal of this study is to utilize paper incineration ash as a cementitious material by reducing the heavy metal content in this processed material by using an advanced removal method. To confirm the success for the research goal, heavy metal content in treated ash is compared to the standard value in waste management laws of Korea.

## Materials and methods

**Figure 1:**
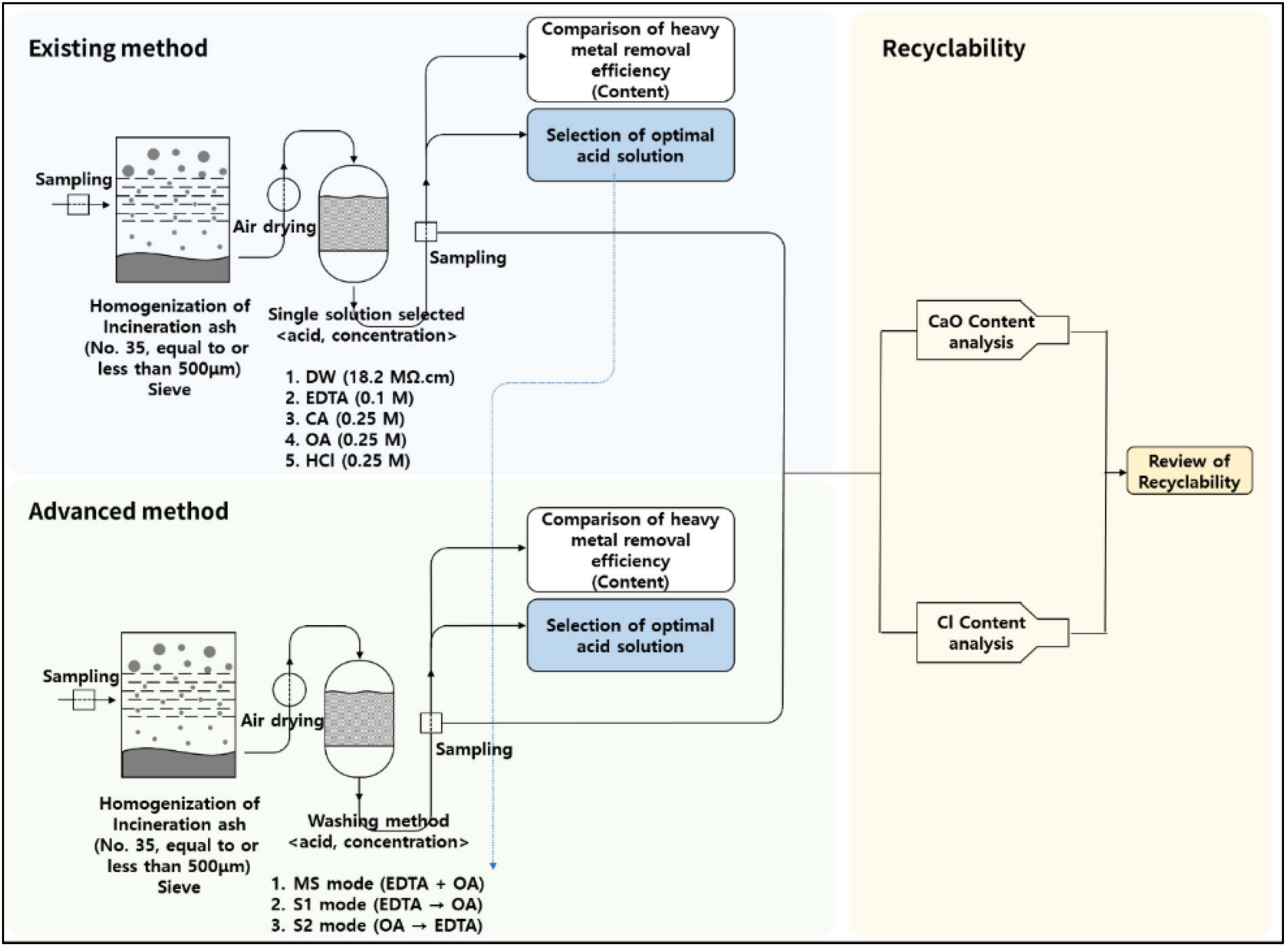
Schematic description of the removal of heavy metals from paper incineration ash.

The process of the removal of heavy metals from paper incineration ash is described in Fig. 1. In the first step, a small-sized sample of incineration ash was obtained using a sieve, and the agent solution was added to the obtained sample. The resulting solution was stirred. Subsequently, it was filtered, and the filtrate of the treated sample was dried. The contents of heavy metals, CaO, and Cl in the treated sample were analyzed. Finally, a recyclability assessment of each sample type was carried out. The detailed methods of each step are described in the following sections.

### Sampling

Using the conical quartering method, incineration ash was collected from a semidry reactor at a paper mill located in Dae-gu, Republic of Korea. After collection, foreign substances, such as small stones, were removed, and particles with diameters ≥ 5 mm were crushed and filtered through a 500-μm standard sieve. The uniform-sized particles were then air-dried for 7–10 days in a shady environment and stored for later use. The stored samples were only removed immediately before the heavy metal removal experiments.

### Removal of heavy metal species from incineration ash

EDTA (99.0%, Reagents Duksan, Korea), OA (99.5%, Reagents Duksan, Korea), HCl (37.0%, Aldrich, USA), distilled water (DW; 18.2 MΩ.cm, ELGA Purelab chorus 1 complete, UK), and CA (99.5% Reagents Duksan, Korea) were used as solutions for the removal of the heavy metals present in the incineration ash. The acid solutions were fixed at a concentration of 0.25 M; however, the EDTA solution was set to 0.1 M owing to its low solubility in water^47^. Furthermore, the incineration ash and each acid solution were mixed at 1:10 (wt. to vol. ratio) and stirred for 3 h to realize heavy metal removal. To verify the effect of the acid solution on the heavy metal removal process, DW was used as the control solution (Table 1). Mixing of the solution including the incineration ash was performed in a 2-L beaker at 400 rpm. The conditions for the treatments are important to determine the removal efficiency and process cost. Various studies have reported on optimized conditions for the concentration of agent in solution, the ratio of ash to solution, and the mixing rate^42,48–53^. Even so, we ensured that each experimental condition was near the end points that reached the maximum removal of heavy metals based on a literature survey^42,48–53^.

**Table 1:** Experimental conditions for the treatment of incineration ash with various agents for heavy metal removal.

Agent solutions	Mixing ratio^a^	Concentration (M)	Mixing time (h)
DW	1:10	–	3
EDTA	1:10	0.1	3
OA	1:10	0.25	3
CA	1:10	0.25	3
HCl	1:10	0.25	3

^a^Mixing ratio of paper sludge ash to solution.

Furthermore, to improve the heavy metal removal efficiency, the advanced removal method, which involved the use of EDTA and OA as agents, was considered. Three modes of this advanced heavy metal removal method were investigated. In the first mode, a mixed solution was used to realize heavy metal removal (marked as MS mode). In the second mode, the EDTA solution was used in the first step, followed by the use of OA solution in the second step (marked as S1 mode), and last, in the third mode, the OA solution was used in the first step followed by the use of EDTA solution in the second step (marked as S2 mode). The mixing ratio of incineration ash to solution, which included the agents, was fixed at 1:5 (wt. to vol.). Furthermore, the mixing time for the MS mode was fixed at 3 h. However, for modes S1 and S2, which both involved the use of single solutions in sequence, the mixing time was fixed at 1.5 h for each step (Table 2). In the MS mode, the samples that were recovered using filtration after mixing were dried at 100 °C for 12 h. In the S1 and S2 modes, the sample that was recovered using filtration in the first step was dried at 100 °C for 12 h before undertaking the second step. After the second step, the same procedure was used to recover the samples. All heavy metal removal treatments were performed for the three parallel samples to assess the repeatability of the experiments, and the relative standard deviation was found to be less than 6%.

**Table 2:** Experimental conditions corresponding to the different modes of the advanced removal method for heavy metal removal from paper sludge ash.

Method	Agents	Mixing ratio^a^	Concentration (M)	Mixing time (h)
MS mode^b^	EDTA, OA	1:5, 1:5	0.1, 0.25	3.0
S1 mode^c^	Step 1: EDTA	1:5	0.1	1.5
	Step 2: OA	1:5	0.25	1.5
S2 mode^d^	Step 1: OA	1:5	0.25	1.5
	Step 2: EDTA	1:5	0.1	1.5

^a^Mixing ratio of paper sludge ash and solution.

^b^Use of EDTA and OA simultaneously.

^c^First step: EDTA; second step: OA.

^d^First step: OA; second step: EDTA.

### Elution tests on incineration ash

Incineration ash (100 g) was accurately weighed and placed in a 2,000 mL Erlenmeyer flask, followed by the addition of distilled water until the sample:solution ratio was 1:10 (wt. to vol.). Thereafter, this prepared solution was continuously shaken for 6 h at 25 °C and atmospheric pressure using a shaker with a shaking frequency and amplitude of approximately 200 rotations per minute and 4–5 cm, respectively. After shaking, the solution was filtered through 1.0-μm glass fiber filter paper, and an appropriate amount of the filtrate was collected for the elution test.

### Analysis methods

#### Analysis of the heavy metal content of incineration ash

The Cu, Pb, As, and Cd contents of the incineration ash were determined using inductively coupled plasma-optical emission spectroscopy (ICP–OES, Optima 2100DV, PerkinElmer, USA). Then, 2.5 mL of HNO_3_ (70%, Aldrich) and 10 mL of HCl (37%, Aldrich) were added to 0.1 g of sample. This solution was heated at 60 °C for 15 min and then cooled. The treated sample was recovered using filter paper with a pore diameter of 0.1 μm. The recovered sample was added to 5 mL of HCl and then heated at 90 °C for 30 min and cooled. DW was added to the samples until the total volume was 100 mL, at which point ICP–OES was carried out. Using the concentrations of the metals based on the measured values, the concentrations of the different heavy metals in the samples after removal were determined according to the following equation:

$$\mathrm{Metal\,concentration\,in\,sample\,}(\mathrm{mg}/\mathrm{kg}) =\frac{({C}_{1}-{C}_{0})}{{W}_{d}}\times \int \times \mathrm{V}$$

where *C*_1_ is the metal concentration in the analytical sample (mg/L); *C*_0_ is the metal concentration in the blank test solution (mg/L); ∫ is the dilution factor; V is the volume of sample container (L).

The Hg content of the solution was determined using a Direct Mercury Analyzer I (DMA-80, Milestone, USA). Specifically, 0.01–1 g of the ash sample was placed in a container. This was followed by drying and pyrolysis to separate the atomized mercury and heating again to a high temperature to propel the atomized mercury into the absorption cell. The functioning of the mercury analyzer is based on the principle of thermal decomposition, amalgamation, and atomic absorption. The mercury in the test sample was released through thermal decomposition by heating to 950 °C and was selectively captured via gold amalgamation. Thereafter, the total mercury in the sample was quantified via atomic absorption spectrophotometry.

To determine the hexavalent Cr (Cr^6+^) content of the ash samples, 2.5 g of the sample was placed in a 250 mL decomposition flask, and then, 50 mL of decomposition solution was added followed by 0.4 g of magnesium chloride (anhydrous) and 0.5 mL of a phosphate buffer solution (0.1 M). The mixture was stirred for 5 min, after which it was filtered through 0.45-μm filter paper and adjusted to a pH of 7.5 using nitric acid (5 M). Then, 95 mL of this pretreated solution was mixed with 2 mL of diphenylcarbazide solution (0.5%) and shaken, and the pH of the sample solution was again adjusted to 2.0 using sulfuric acid (20%). Thereafter, a portion of the solution was placed in a 10-mm absorption cell, and the Cr^6+^ content of the solution was measured using a UV–visible Spectroscopy System (Agilent 8453, Agilent Technologies, USA).

#### Analysis of the possibility of recycling incineration ash

The concentrations of Pb, Cu, Cd, As, Hg, Cr^6+^, and Cl in the eluted solution of the original sample and in the treated samples were investigated in regard to the recycling possibility of the incineration ash. The concentrations of Pb, Cu, Cd, and As were measured via ICP–OES analysis. The concentrations of Hg and Cr^6+^ were measured using a UV–visible light spectroscopy system. The concentration of Cl was also investigated even though it is not a heavy metal, because the Cl concentration must be controlled as per the waste management laws of Korea. The concentration of Cl was calculated using ion chromatography (ICS-2000, Dionex, USA). In addition, to assess the change in the composition of the incineration ash sample before and after heavy metal removal, measurements were performed using an X-ray fluorescence spectrometer (XRF, EPSILON 4, PANalytical, NLD). Before the XRF analysis, the incineration ash samples were calcined at 700 °C to remove organic matter and achieve thermal stability.

## Results and discussion

### Effect of various agents on heavy metal removal efficiency

Table 3 shows the heavy metal contents of the paper incineration ash before and after extraction with the various agents. From this table, it is evident that the original paper incineration ash samples had a high heavy metal content, which varied in the order: + Pb > Cu > Cd > As > Hg; Cr^6+^ was not detected. It was also observed that volatile heavy metals, such as Pb and Cd, tended to vaporize and recondense on fine particles during the incineration procedure. This explains the increased contents of these species in the fly ash. Additionally, high Pb, Cu, and As contents were observed when the incinerated material was derived from paper waste, including synthetic resin^54^. It was also observed that all the agents brought about a decrease in the Pb and Cu contents of the incineration ash. Specifically, the Pb content of the incineration ash following removal using a single agent was in the order DW > CA > HCl > OA > EDTA. In the case of Cu, the order was DW > HCl > CA > EDTA > OA. Furthermore, both Pb and Cu showed the lowest contents in the resulting incineration ash when EDTA and OA were used as the extraction agents. For the As content, the order was DW ≈ CA ≈ HCl > EDTA > OA, confirming that the As was not removed when HCl and CA were used. Furthermore, the Hg contents of the treated incineration ash samples were similar regardless of the agent type that was used. The Cd content of the samples followed the order DW > CA > HCl > EDTA > OA.

**Table 3:** Comparison of heavy metal contents before and after treatment using various agent solutions.

Heavy metal	Unit	Original sample	Agent solutions				
			Distilled water	EDTA	CA	OA	HCl
Pb	mg/kg	944.70 ± 40.62	900.00 ± 45.00	750.02 ± 28.50	890.01 ± 35.69	820.01 ± 38.54	860.02 ± 33.20
Cu		902.40 ± 37.90	900.30 ± 38.71	790.10 ± 27.65	830.23 ± 35.12	706.09 ± 31.77	860.12 ± 26.84
As		6.72 ± 0.26	6.72 ± 0.25	6.32 ± 0.20	6.72 ± 0.24	5.40 ± 0.29	6.70 ± 0.18
Hg		0.10 ± 0.01	0.09 ± 0.01	0.09 ± 0.01	0.08 ± 0.01	0.09 ± 0.01	0.10 ± 0.01
Cd		17.48 ± 0.68	17.20 ± 0.89	16.10 ± 0.66	16.40 ± 0.56	15.90 ± 0.73	16.30 ± 0.54
Cr^6+^		ND^a^	ND	ND	ND	ND	ND

^a^Not detected.

**Figure 2:**
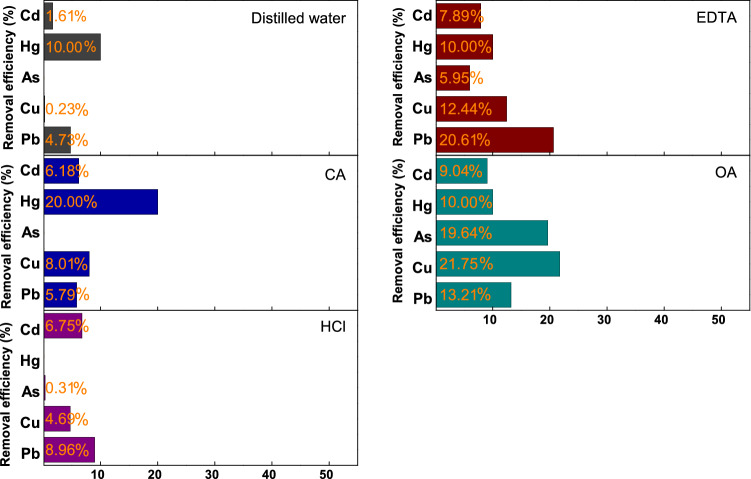
Comparison of the heavy metal removal efficiencies of various solutions.

The heavy metal removal efficiency of each agent solution was closely examined, and the results obtained are shown in Fig. 2, from which it is evident that DW showed the lowest removal efficiency for all the heavy metals. When OA was used, the Cu, As, and Cd removal efficiencies were 21.75, 19.64, and 9.04%, respectively, which were the highest removal efficiencies for each of these metals. EDTA showed the highest Pb removal efficiency at 20.61%. CA exhibited removal efficiencies that were less than 10% for all the heavy metals except Hg (20%). However, given the very low Hg content of the incineration ash, it was difficult to accurately compare the removal efficiencies of the various single-agent solutions. Furthermore, HCl displayed removal efficiencies below 10% for all heavy metals. Thus, EDTA and OA, which exhibited relatively higher removal efficiencies for the heavy metals, were selected as two agents for the advanced removal method, which was performed in three modes, to realize heavy metal removal from the incineration ash. The pH of each solution was measured by a pH meter, as shown in Table 4. The pH of each solution with the addition of the different agents was ordered as HCl < OA < CA < EDTA < DW. However, when the paper incineration ash samples were added to the agent solutions, the pH values of the resulting solutions were similar and ranged between 12–13; this may be due to the strong basic properties of the ash samples. Although pH is an important factor for the ionization of heavy metals during the removal process, the effect of pH might be negligible in the present study.

**Table 4:** The measured pH values of the various solutions.

Agent solutions								
Condition	Distilled water	EDTA	CA	OA	HCl	MS mode^a^	S1 mode^b^	S2 mode^c^
Solution agent	7.52	4.54	1.95	1.18	1.17	1.46	4.54	1.18
							1.18	4.54
Solution including ash	13.10	12.30	12.50	12.12	11.96	12.00	12.50	12.30
							12.43	12.45

^a^Removal using EDTA and OA simultaneously.

^b^First step: EDTA; second step: OA.

^c^First step: OA; second step: EDTA.

### Advanced removal method for heavy metal removal using EDTA and OA

#### Effect of removal methods on heavy metal removal efficiency

To improve the efficiency of heavy metal removal from paper incineration ash, an advanced removal method involving the use of two agents was considered. EDTA and OA were selected as the agents owing to their high efficiencies in the removal of Pb and Cu, As, and Cd, in order. To optimize the advanced removal method, the performances of the EDTA and OA removals were tested under the three different modes (MS, S1, and S2). The results obtained are shown in Table 5. From this table, it is evident that the advanced removal methods showed better heavy metal removal effects than the removal modes involving the use of single-agent solutions. This observation could be attributed to the fact that EDTA and OA each complemented their lower individual heavy metal removal efficiencies. The Pb content of the treated incineration ash samples was in the order of OA (820.01 mg/kg) > EDTA (750.02 mg/kg) > S1 mode (534.60 mg/kg) > S2 mode (516.96 mg/kg) > MS mode (480.03 mg/kg). Furthermore, the Cu content of the incinerated ash following the removals was in the order of EDTA (790.10 mg/kg) > OA (706.09 mg/kg) > S1 mode (670.00 mg/kg) > S2 mode (648.40 mg/kg) > MS mode (620.01 mg/kg). The As content of the treated samples, which showed a tendency that was similar to that of Cu, given that Cu could adsorb As^39^, varied in the order of EDTA (6.32 mg/kg) > OA (5.40 mg/kg) > S1 mode (5.38 mg/kg) > S2 mode (4.92 mg/kg) > MS mode (4.81 mg/kg). All the removals resulted in ash samples with a similar Hg content (approximately 0.10 mg/kg). The Cd content of the incineration ash following the different removals was in the order of EDTA (16.10 mg/kg) > OA (15.90 mg/kg) > S1 mode (15.62 mg/kg) > S2 mode (15.50 mg/kg) > MS mode (15.40 mg/kg), which is similar to the order of contents for other heavy metals. These results indicate that the MS mode resulted in the lowest heavy metal contents owing to the synergistic action of EDTA and OA. The synergistic action of EDTA and OA is caused by the different removal mechanisms of EDTA and OA. The functional groups of EDTA remove the heavy metals that exist in their metallic phases. In contrast, OA extracts heavy metal oxides by reduction^39^. In addition, complexes of OA and metal oxides can prevent the formation of metal oxides^55^. Therefore, the simultaneous use of EDTA and OA enhanced the removal efficiency owing to the synergistic effects of EDTA and OA on the removal of heavy metals.

**Table 5:** Comparison of the heavy metal contents of paper incineration ash following treatment using various removal methods.

Heavy metal	Unit	Original sample	Removal methods				
			Existing methods		Advanced methods		
			EDTA	OA	MS mode^a^	S1 mode^b^	S2 mode^c^
Pb	mg/kg	944.70 ± 40.62	750.02 ± 28.50	820.01 ± 38.54	480.03 ± 22.61	534.60 ± 28.76	516.96 ± 14.27
Cu		902.40 ± 37.90	790.10 ± 27.65	706.09 ± 31.77	620.01 ± 9.92	670.00 ± 18.83	648.40 ± 28.66
As		6.72 ± 0.26	6.32 ± 0.20	5.40 ± 0.29	4.81 ± 0.15	5.38 ± 0.12	4.92 ± 0.21
Hg		0.10 ± 0.01	0.09 ± 0.01	0.09 ± 0.01	0.09 ± 0.01	0.09 ± 0.01	0.08 ± 0.01
Cd		17.48 ± 0.68	16.10 ± 0.66	15.90 ± 0.73	15.40 ± 0.47	15.62 ± 0.33	15.50 ± 0.67
Cr^6+^		ND^d^	ND	ND	ND	ND	ND

^a^Use of EDTA and OA simultaneously.

^b^First step: EDTA; second step: oxalic acid.

^c^First step: oxalic acid; second step: EDTA.

^d^Not detected.

**Figure 3:**
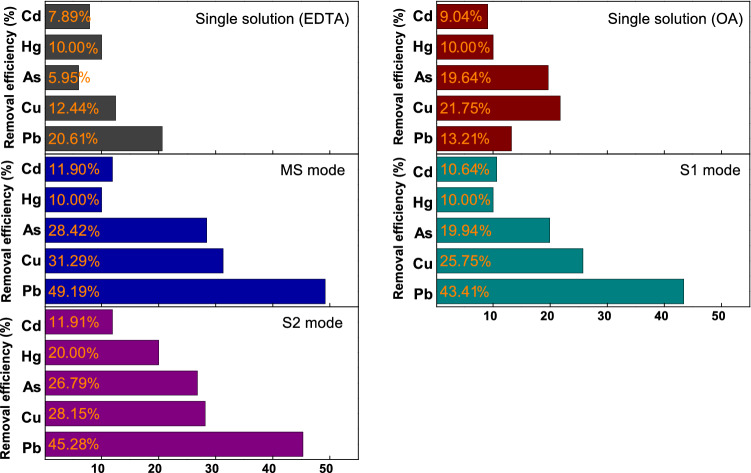
Comparison of the efficiencies of the different heavy metal removal methods.

The heavy metal removal efficiencies of the existing removal methods as well as those corresponding to the three modes of the advanced removal method are shown in Fig. 3, from which it is evident that the MS mode exhibited the highest removal performance for all the heavy metals (Pb = 49.19%, Cu = 31.29%, As = 28.42%, Cd = 11.90%), except for Hg. It appeared that removal using a mixture of EDTA and OA not only improved the chelating action of EDTA with heavy metals but also made the solution pH suitable for the elution of heavy metals^42^. The comparison of the removal efficiencies of the different removals with respect to Hg removal was challenging given the very low Hg content of the paper incineration ash.

#### Examination for the possibility of recycling and utilizing treated incineration ash

The amount of eluted heavy metals as well as the amount of eluted chlorine are important indicators of the possibility of recycling and utilizing treated incineration ash because the eluted heavy metals pose a serious problem to the environment and human health^56^. Furthermore, chlorine contaminates water and soil, finally hindering plant growth and rendering water unsuitable for drinking^57^. Table 6 shows the amount of eluted heavy metals and chlorine obtained following the removal of incineration ash using various removal modes and the specified elution conditions. In the case of incineration ash treated using distilled water (3.47 mg/L), CA (3.43 mg/L), and HCl (3.32 mg/L), the eluted Pb exceeded the standard (1.00 mg/L). The amount of Pb eluted following the different removals was in the order of OA (0.87 mg/L) > EDTA (0.73 mg/L) > S1 mode (0.63 mg/L) ≈ S2 mode (0.61 mg/L) > MS mode (0.54 mg/L). It was also observed that the amount of Pb eluted was small when its content in the treated incineration ash was low. Likewise, the amount of Cu eluted was also dependent on its content in the treated incineration ash, varying in the order of distilled water (0.22 mg/L) > CA (0.04 mg/L) = EDTA (0.04 mg/L) ≈ OA (0.03 mg/L) = S1 mode (0.03 mg/L) = S2 mode (0.03 mg/L) ≈ MS mode (0.02 mg/L). All the treated incineration ashes, regardless of agent solutions, met the standard for the elution of Cu. However, even though the Cu and Pb contents of the incineration ash were similar, a smaller amount of Cu was eluted. This finding could be attributed to the differences in the desorption capacity of heavy metals^58^. All the removals resulted in As, Hg, Cd, Cr^6+^, and Cl elution concentrations below the detection limit. Furthermore, a large amount of Cl was eluted from the original sample, while the treated samples did not show any Cl elution. It is believed that this is because most of the chlorine was removed following dissolution in water, and possibly, the acidic solution further dissolved insoluble chlorides^59,60^. The paper incineration ash treated by the advanced removal method met the standard value for the recycling of ash (shown in Table 6) based on the waste management laws of Korea. CaO and Cl contents are important factors that determine the recyclability of treated incineration ash, where the solidification effect of this product is greater when its CaO content is high^61^. Conversely, the presence of Cl hinders its solidification effect; thus, the removal of Cl is necessary^62^. After removal, there was an increase in the CaO content of the incineration ash from 70% to 77–79%, while its Cl content, which was high at 16.74%, decreased to less than 2% after extraction in all the samples, as shown in Table 6. Because the CaO content of ordinary Portland cement includes 60–70% CaO^63^, the treated paper incineration ash can be utilized as a supplementary cementitious material. This increase in the CaO content of the incineration ash after removal possibly resulted from the increase in the relative content of CaO as Cl was removed during the removal process. Thus, the incineration ash treated using the advanced removal method showed suitability for application as a supplementary cementitious material regardless of the sequence of EDTA and OA removal steps. In addition, the XRF results of the paper incineration ash are summarized in Table 7. Among the components, the amount of CaO was the highest. The components of metals in the ash increased when the ash was treated using various single-agent solutions, while the components of alkaline metals (Na, K) and halogens (Br, Cl) decreased. This result was ascribed to the fact that alkaline metals and halogens can be easily dissolved by water^64^. However, in further studies, it would be necessary to investigate the effects of removal conditions, such as the temperature, exposure time, and concentration of the removal agent in solution, to optimize their removal from incineration ash.

**Table 6:** The results of the recyclability assessment for the paper incineration ash following removal using various removal methods.

Indicators for recyclability	Element	Unit	Content of original sample	Standard	Removal methods							
					Existing methods					Advanced methods		
					Distilled water	CA	HCl	EDTA	OA	MS mode^a^	S1 mode^b^	S2 mode^c^
Concentration	Pb	mg/L	3.64 ± 0.13	1.00	3.47 ± 0.12	3.43 ± 0.12	3.32 ± 0.11	0.73 ± 0.03	0.87 ± 0.04	0.54 ± 0.03	0.63 ± 0.02	0.61 ± 0.02
	Cu		0.22 ± 0.01	1.00	0.22 ± 0.01	0.04 ± 0.01	0.21 ± 0.01	0.04 ± 0.01	0.03 ± 0.01	0.02 ± 0.01	0.03 ± 0.01	0.03 ± 0.01
	As		0.06 ± 0.01	0.50	ND^d^	ND	ND	ND	ND	ND	ND	ND
	Hg		0.10 ± 0.01	0.0030	ND	ND	ND	ND	ND	ND	ND	ND
	Cd		0.10 ± 0.01	0.10	ND	ND	ND	ND	ND	ND	ND	ND
	Cr^6+^		0.05 ± 0.01	0.10	ND	ND	ND	ND	ND	ND	ND	ND
	Cl		1,261.36 ± 53.73	250	ND	ND	ND	ND	ND	ND	ND	ND
Content	CaO	%	70.02	60–70	79.05 ± 3.20	79.32 ± 2.54	77.12 ± 3.18	77.99 ± 3.89	77.12 ± 3.18	78.02 ± 3.92	79.05 ± 3.20	78.32 ± 3.38
	Cl		16.74	–	1.48 ± 0.07	1.91 ± 0.07	1.76 ± 0.08	1.8 ± 0.07	1.76 ± 0.08	1.68 ± 0.08	1.48 ± 0.07	1.54 ± 0.08

^a^Removal using EDTA and OA simultaneously.

^b^First step: EDTA; second step: OA.

^c^First step: OA; second step: EDTA.

^d^Not detected.

**Table 7:** The XRF results for the paper incineration ash following removal using various removal methods.

Element	Unit	Content of Original sample	Removal methods					
			Existing methods			Advanced methods		
			Distilled water	EDTA	OA	MS mode^a^	S1 mode^b^	S2 mode^c^
CaO	%	70.02	79.05	77.99	77.12	78.02	79.05	78.32
Cl		16.74	1.48	1.87	1.76	1.68	1.48	1.54
SiO_2_		2.89	4.98	4.89	5.68	5.64	5.55	5.87
MgO		2.04	3.58	3.46	4.11	3.87	4.01	4.13
Fe_2_O_3_		1.38	2.26	2.2	2.73	2.47	2.26	2.25
TiO_2_		0.92	1.24	1.14	1.61	1.66	1.45	1.46
Al_2_O_3_		0.81	1.38	1.37	1.96	1.79	1.81	1.94
ZnO		0.75	1.21	1.16	1.56	1.42	1.45	1.44
Na_2_O		0.52	–	0.29	–	0.36	0.1	0.3
K_2_O		0.35	0.11	0.12	0.26	0.21	0.27	0.22
NiO		0.28	0.48	0.45	0.71	0.62	0.52	0.52
BaO		0.22	0.5	0.2	0.9	0.9	0.8	0.8
Br		0.16	0.02	0.03	0.02	0.04	0.03	0.03
PbO		0.15	0.21	0.2	0.41	0.31	0.26	0.26
CuO		0.13	0.22	0.22	0.31	0.24	0.24	0.18
MnO		0.13	0.22	0.22	0.34	0.26	0.27	0.26
Sb_2_O_3_		0.12	0.17	0.16	0.16	0.18	0.14	0.14
P_2_O_5_		0.07	0.13	0.12	0.13	0.13	0.13	0.13
SrO		0.07	0.06	0.06	0.08	0.06	0.04	0.07
Cr_2_O_3_		0.05	0.08	0.07	0.08	0.07	0.08	0.08
ZrO_2_		0.04	0.07	0.06	0.07	0.07	0.06	0.06

^a^Removal using EDTA and OA simultaneously.

^b^First step: EDTA; second step: OA.

^c^First step: OA; second step: EDTA.

## Conclusion

In this study, heavy metals in incineration ash were removed using various single-agent solutions, i.e., EDTA, OA, CA, and HCl. EDTA showed the highest removal efficiency with respect to Pb, while OA showed the highest removal efficiencies with respect to Cu, As, and Cd. The advanced removal method, which involved the use of EDTA and OA in three different modes, resulted in improved heavy metal removal efficiencies. Specifically, the mode involving the simultaneous use of EDTA and OA exhibited the best removal efficiencies for all the heavy metals owing to the synergistic actions of EDTA and OA when they coexist in solution (removal efficiency: Pb = 49.19%, Cu = 31.29%, As = 28.42%, Cd = 11.90%). Furthermore, to examine the possibility of recycling and utilizing the treated incineration ash, elution tests were performed, and the CaO and Cl contents of the treated samples were measured. The eluted amounts of heavy metals and chlorine decreased when the incineration ash was treated using the advanced removal methods. It was also confirmed that the CaO content of incineration ash increased, while its chlorine decreased when the three modes of the advanced removal method were applied to remove the heavy metals from the incineration ash. Thus, the advanced removal method is a promising strategy by which heavy metals can be removed from paper incineration ash to obtain a recyclable product. However, the effect of treatment conditions, such as the concentration of the agents, treatment time, and treatment temperature, should be investigated to optimize the process efficiency in future work.

## Data availability

All data and materials supporting the conclusion of this study can be found in the manuscript.
